# DISCERNING STATUS: SUBJECTIVE SOCIAL POSITION AND COGNITIVE HEALTH AMONG OLDER ADULTS IN RURAL SOUTH AFRICA

**DOI:** 10.1093/geroni/igz038.3008

**Published:** 2019-11-08

**Authors:** Lindsay C Kobayashi, Guy Harling, Meagan T Farrell, Ryan G Wagner, Lisa F Berkman

**Affiliations:** 1 Georgetown University, Washington, District of Columbia, United States; 2 University College London, London, United Kingdom; 3 Harvard Center for Population and Development Studies, Cambridge, Massachusetts, United States; 4 University of the Witwatersrand, Johannesburg, South Africa

## Abstract

Rapid population aging in high absolute poverty settings, such as much of South Africa, demands new research on the social context factors that affect cognitive aging in these settings. We investigated the relationships between subjective social position within one’s village and cognitive function and impairment, with the rationale that psychosocial stress induced by low relative social position may affect cognitive aging outcomes independently of absolute socioeconomic conditions. Data were from the population-representative HAALSI study of 5,059 adults aged 40+ in rural Agincourt, South Africa. Subjective social position was assessed using the MacArthur Network social ladder, which asks respondents to indicate how high up a ladder they stand, relative to others, in their village. Cognitive function was a composite z-score of time orientation and word recall tests; scores ≤1.5 standard deviations (SD) below the mean indicated cognitive impairment. Twenty percent of those on the bottom rung had cognitive impairment, declining to 2% on the top rung. In regression models adjusted for age, sex, country of birth, education, literacy, marital status, employment, and asset-based household wealth, each ladder rung increase was associated with an 0.05 SD increase in cognitive z-score (95% CI: 0.04-0.06), and a 17% decrease in odds of cognitive impairment (OR=0.83; 95% CI: 0.79-0.88); these associations were greater in magnitude than those for years of education with these cognitive outcomes. Future work should examine if these relationships persist longitudinally, and investigate the mechanisms of these relationships, including village-level contextual factors that may contribute to subjective social position in this setting.

